# Clinical Efficacy and Safety of Yellow Oil Formulations 3 and 4 versus Indomethacin Solution in Patients with Symptomatic Osteoarthritis of the Knee: A Randomized Controlled Trial

**DOI:** 10.1155/2020/5782178

**Published:** 2020-07-25

**Authors:** Nut Koonrungsesomboon, Supanimit Teekachunhatean, Sunee Chansakaow, Nutthiya Hanprasertpong

**Affiliations:** ^1^Department of Pharmacology, Faculty of Medicine, Chiang Mai University, Chiang Mai 50200, Thailand; ^2^Musculoskeletal Science and Translational Research Center, Chiang Mai University, Chiang Mai 50200, Thailand; ^3^Center of Thai Traditional and Complementary Medicine, Faculty of Medicine, Chiang Mai University, Chiang Mai 50200, Thailand; ^4^Department of Pharmaceutical Sciences and Medicinal Plant Innovation Center, Faculty of Pharmacy, Chiang Mai University, Chiang Mai 50200, Thailand

## Abstract

**Background:**

Topical nonsteroidal anti-inflammatory drugs (NSAIDs) are widely prescribed for the treatment of symptomatic osteoarthritis (OA) of the knee. However, searching for alternatives such as locally available medicinal herbs to manage OA knee pain remains of clinical value. The objective of the present study was to evaluate the efficacy and safety of two yellow oil formulations in patients with OA of the knee.

**Methods:**

This prospective, randomized, single-blind, active-controlled, noninferiority study enrolled 102 patients with OA of the knee. Eligible patients were randomly assigned to apply either yellow oil formulation 3 (YOF3), yellow oil formulation 4 (YOF4), or indomethacin solution (INDO) topically four times daily for four weeks. Outcomes were assessed on a biweekly basis. The primary efficacy outcome measure was a 100 mm visual analog scale (VAS) of pain, while secondary endpoints included knee function, physical performance assessments, and safety parameters. Modified intention-to-treat and per-protocol analyses were applied. Assessment of noninferiority was done with a prespecified margin of 10 mm for VAS pain.

**Results:**

Of 102 patients enrolled, 86 completed the study: 29/34 in the YOF3 group, 25/34 in the YOF4 group, and 32/34 in the INDO group. The absolute reduction in VAS pain at the final evaluation was −25.06 ± 13.91, −18.50 ± 16.06, and −23.38 ± 10.05 mm in the YOF3, YOF4, and INDO groups, respectively (*p*=0.169). Only YOF3 was found to be noninferior to INDO. Other efficacy outcomes were significantly improved in all three groups. All the interventions were well tolerated; no skin rash was observed in any of the three groups.

**Conclusions:**

YOF3 was shown to be noninferior to INDO in relieving knee pain and should be considered an alternative for the treatment of symptomatic OA of the knee. Further research into the mechanism of action of YOF3 and its long-term efficacy and safety is required.

## 1. Introduction

Osteoarthritis (OA) is a chronic degenerative disease characterized by a progressive loss of articular cartilage [1]. OA may occur in any joint but most commonly affects the knee, causing pain and functional disability [2]. OA of the knee poses a huge burden on patients and the healthcare system, and it is one of the leading causes of disability globally [3]. In general, females are more commonly affected with knee OA than males, and the incidence of knee OA continues to increase with advanced age and obesity [4]. By age 85, nearly half of the people may develop symptomatic OA of the knee, requiring pharmacological management for adequate pain control [5].

Nonsteroidal anti-inflammatory drugs (NSAIDs) are the fundamental pharmacological management tools for pain and inflammation in patients with OA of the knee [6]. Topical forms of NSAIDs offer the advantage over oral preparations of reduced systemic adverse effects while providing adequate pain relief at the knee joints in individuals with localized mild to moderate pain [7]. Various lines of evidence support the clinical efficacy of topical NSAIDs in reducing pain and stiffness as well as in improving the joint function of knee OA [8–10]. At present, major international clinical practice guidelines consistently recommend the use of topical NSAIDs as an initial therapeutic option for the treatment of symptomatic OA of the knee, particularly among elderly patients [11–14].

Although topical NSAIDs are widely used for pain relief, their relatively high cost encourages a search for alternatives such as locally available medicinal herbs historically used in traditional folk medicine. In the past few decades, indigenous knowledge of traditional and herbal medicines has contributed significantly to modern therapeutic drugs. For example, several recent studies have provided a scientific basis for the use of herbal medicinal products in the management of pain in symptomatic OA of the knee [15–19]. In Thailand, there are a number of locally available medicinal herbs which possess anti-inflammatory and analgesic properties [20]. The yellow oil or the hot oil extract from *Zingiber montanum* Koenig (*Z. cassumunar* Roxb.) is one of those herbs that have been used to treat musculoskeletal disorders, thanks to its anti-inflammatory and analgesic properties based on indigenous knowledge which is passed from generation to generation in traditional folk medicine. In this study, we aimed to evaluate the efficacy and safety of two yellow oil formulations–yellow oil formulation 4 (YOF4), which is a formulation included in the Thai National List of Essential Medicines, and its modified formulation, that is, yellow oil formulation 3 (YOF3) in patients with OA of the knee by means of a randomized controlled trial. Identifying effective and safe therapeutic herbal formulations to manage osteoarthritic pain is concordant with the priorities of the Thailand 4.0 policy. This policy is an economic model aimed at moving the country toward stability, prosperity, and sustainability.

## 2. Methods

This study followed the Osteoarthritis Research Society International (OARSI) Clinical Trials Recommendations for the design, conduct, and reporting of clinical trials for OA of the knee [21]. It was conducted in accordance with the Declaration of Helsinki [22]. The study protocol and related materials were approved by the Research Ethics Committee of the Faculty of Medicine, Chiang Mai University (461/2560). The trial was prospectively registered with the Thai Clinical Trials Registry (TCTR20171219003). Written informed consent was obtained from all patients prior to their participation in the trial.

### 2.1. Study Design and Setting

**Figure 1:**
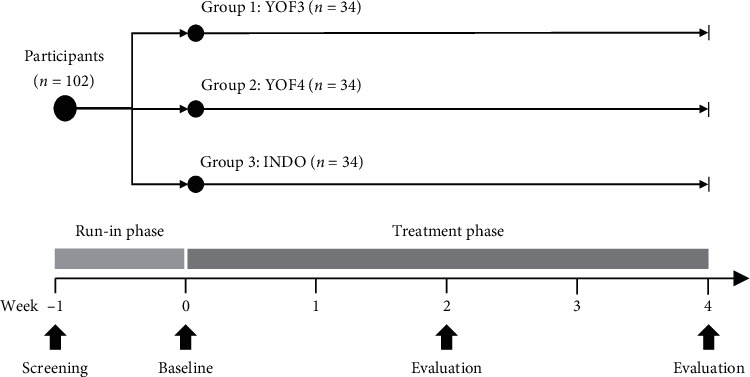
Study design.

This prospective, randomized, single-blind, parallel-group, three-arm, active-controlled, noninferiority study was conducted at the Faculty of Medicine, Chiang Mai University, Chiang Mai, Thailand. The trial consisted of a run-in phase of one week and a four-week treatment phase. Enrolled patients were asked to visit the study site for outcome assessment every two weeks during the study (Figure 1).

### 2.2. Study Participants

Patients aged ≥45 years with primary OA of the knee for more than three months were recruited. The diagnosis of OA of the knee was based on the American College of Rheumatology's clinical and radiographic criteria [23]. Patients were eligible for inclusion if they had a visual analog scale (VAS) pain score of 35–75 mm at baseline and were able to walk. Patients were excluded if they had any of the following conditions: another underlying arthritis (e.g., rheumatoid arthritis or gouty arthritis), hyperuricemia (serum uric acid >9 mg/dL), condition requiring knee surgery in the next few months, use of intra-articular corticosteroid injections in the past three months, use of symptomatic slow-acting drugs for OA (SYSADOA) (e.g., glucosamine sulfate or chondroitin sulfate) within the previous four months or discontinuation of SYSADOA less than six months prior to enrollment, history of allergic reaction to topical NSAIDs or any ingredients in yellow oil formulations, clinically significant skin lesions on the knee, known pregnancy or lactation, or clinically significant abnormalities of blood chemistry or other hematological parameters.

A total of 102 patients were planned to be enrolled in the trial. The sample size of 102 was determined based on a noninferiority margin of 10 [24, 25], assuming a mean difference (MD) of 0 and a standard deviation (SD) of 14 [26]. With a precision and confidence level of 95%, 80% power, and a dropout rate of 25%, 34 patients per treatment group were required [27].

### 2.3. Randomization, Allocation Concealment, and Blinding

Blocked randomization (a block size of 6) was applied to randomly assign eligible patients into one of three groups (1 : 1:1). A computer-generated randomization list was prepared beforehand by research staff. Opaque sealed envelopes containing the list were numbered in advance and opened sequentially by research staff after each patient met the eligibility criteria and underwent randomization. Both outcome assessors and enrolled patients were blinded to the treatment allocation; however, the patients might have been aware of the treatment to which they had been assigned because the three investigational solutions were unalike in color.

### 2.4. Study Interventions

The three investigational solutions used in the trial were yellow oil formulation 3 (YOF3), yellow oil formulation 4 (YOF4), and indomethacin solution (INDO). YOF3 and YOF4 were manufactured at the Thai Traditional Medicine Manufacturing Laboratory, Faculty of Pharmacy, Chiang Mai University. Thin-layer chromatographic method was applied for the purposes of the quality control of yellow oil formulations. YOF3 was composed of dry rhizomes of *Z. montanum* Koenig (*Z*. *cassumunar* Roxb.), dry flowers of *Syzygium aromaticum* Merr. et Perry, dry bark of *Cinnamomum aromaticum* Nees, menthol, racemic camphor, borneol, and sesame oil. YOF4 was composed of fresh rhizomes of *Z. montanum* Koenig (*Z*. *cassumunar* Roxb.), fresh rhizomes of *Curcuma longa* L., dry flowers of *S. aromaticum* Merr. et Perry, coconut oil, menthol, racemic camphor, and methyl salicylate. The components of YOF3 and YOF4 are listed in Table S1. A 1% indomethacin solution (Elmetacin®) purchased from OLIC (Thailand) Limited (1A 708/30) was used as an active control in this trial.

YOF3, YOF4, and INDO were dispensed in identical plastic spray bottles. Patients were instructed to apply three sprays of the intervention (equivalent to an approximate total volume of 0.75 mL) per affected knee, four times a day. The first, second, and third sprays were to be applied to the anteromedial, anterolateral, and posterior aspects of the knee, respectively.

### 2.5. Study Procedures and Outcome Assessments

During the one-week run-in period, eligible patients were instructed to discontinue all other pain relief medications (including NSAIDs and analgesics). At the start of week 0, patients were randomly assigned to receive either YOF3, YOF4, or INDO (1 : 1:1) (Figure 1). Use of any other analgesics, anti-inflammatory drugs (including other NSAIDs), corticosteroids, opioid/opioid derivatives, or other treatment modalities (e.g., herbal products, acupuncture, and massage) was not allowed during the study. Patients were prematurely withdrawn from the trial if they had an exacerbation of severe OA knee pain (pain score >75 mm) requiring other treatment modalities, used other analgesics or anti-inflammatory drugs, had severe allergic reactions to the intervention, or were lost to follow-up.

Outcome assessment was performed at baseline (at the end of the one-week run-in period) and at the end of weeks 2 and 4 (Figure 1). The primary efficacy outcome measure was a horizontal 100 mm VAS assessment of knee pain (VAS pain) over the previous two days (0 = no pain; 100 = worst imaginable pain) [28]. The secondary outcome measures included (i) a horizontal 100-mm VAS assessment of knee joint stiffness (VAS stiffness) over the previous two days (0 = no stiffness and able to move the knee freely; 100 = severe stiffness with very difficult movement), (ii) a 10-step stair climb test (SCT) (time taken to climb up 10 steps) [29], (iii) a timed up and go (TUG) test (time taken to stand up from a chair, walk three meters, turn around, walk back to the chair, and sit down again) [29], (iv) the Knee Injury and Osteoarthritis Outcome Score (KOOS) (five domains: pain frequency and severity during functional activities (nine items), other symptoms (e.g., the severity of knee stiffness and the presence of swelling, grinding, and range of motion restriction) (seven items), difficulty experienced during activities of daily living (17 items), difficulty experienced with sport and recreation activities (five items), and knee-related quality of life (four items)) [30, 31], and (v) a horizontal 100-mm VAS assessment of the patient's and the physician's opinions of overall improvement (0 = no change; 100 = excellent improvement). Physical examination was performed, and nondirective questions were asked at each visit to monitor patient safety and to detect any adverse events. Drug compliance was assessed by measuring the volume of unused solution remaining in the spray bottle. In patients with bilateral OA of the knee, both knees were assessed for drug safety, while the knee with the higher pain score at baseline was used for efficacy assessment.

### 2.6. Statistical Analysis

Analyses of efficacy outcomes were undertaken using the modified intention-to-treat (MITT) and per-protocol (PP) approaches. In the MITT analysis, the last observation carried forward method was used to analyze the data of patients who had prematurely withdrawn from the study and those with drug compliance of <70%. For the safety evaluations, all patients who had received at least one dose of the assigned intervention were analyzed.

Continuous variables are presented as mean ± SD. Within-group comparisons were conducted to determine any differences in the mean values of each variable between baseline and the two consecutive follow-up visits; one-way repeated measures analysis of variance (ANOVA), with the least significant difference (LSD) test, was applied. For between-group comparisons, mean changes from baseline were compared using one-way ANOVA followed by the Dunnett test [32]. Patients were considered to be responders if their VAS pain decreased by at least 50% from the baseline value [33]. Dichotomous variables are reported as frequencies; the chi-squared test was used to determine differences in the percentage of responders among the three groups.

For assessment of noninferiority, a comparison between the YOF3 or YOF4 group and the INDO group on VAS pain was conducted, with a prespecified noninferiority margin of 10 mm [24, 25]. Noninferiority was declared if the upper limit of the two-sided 95% CI for the MD of VAS pain did not exceed a margin of 10 mm.

Statistical analysis was performed using SPSS version 22.0. A *p* value of <0.05 was considered to indicate statistical significance.

## 3. Results

**Figure 2:**
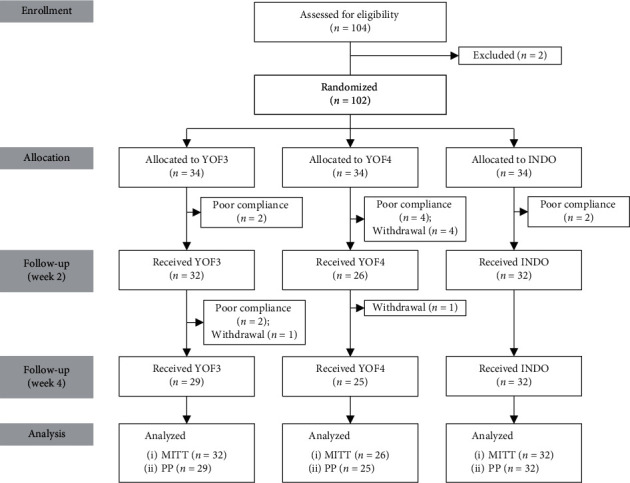
Flow diagram of the progress through all phases of this three-arm, randomized controlled study i.e., enrollment, intervention allocation, follow-up, and data analysis.

Between January 2018 and September 2018, 104 patients with OA of the knee were initially assessed for eligibility, of whom 102 were enrolled and randomly assigned to either the YOF3 group (*n* = 34), the YOF4 group (*n* = 34), or the INDO group (*n* = 34) (Figure 2). The mean age of the enrolled patients was 61.8 ± 7.2 years, 91.2% were female, and 76.5% had OA of both knees. Baseline characteristics of the patients were comparable among the three groups (Table 1). After randomization, six patients prematurely withdrew from the study: four in the YOF4 group during weeks 0–2 (due to increased knee pain, a common cold, a fall with shoulder dislocation, and loss to follow-up), one in the YOF4 group during weeks 2–4 (due to increased knee pain), one in the YOF3 group during weeks 2–4 (due to a brain tumor requiring hospitalization), and none in the INDO group. Ten patients were excluded from the efficacy analysis due to poor compliance: two, four, and two during weeks 0–2 in the YOF3, YOF4, and INDO groups, respectively, and two during weeks 2–4, both in the YOF3 group. A total of 86 patients (84.3%) completed the study: 29 (85.3%) in the YOF3 group, 25 (73.5%) in the YOF4 group, and 32 (94.1%) in the INDO group (Figure 2).

**Figure 3:**
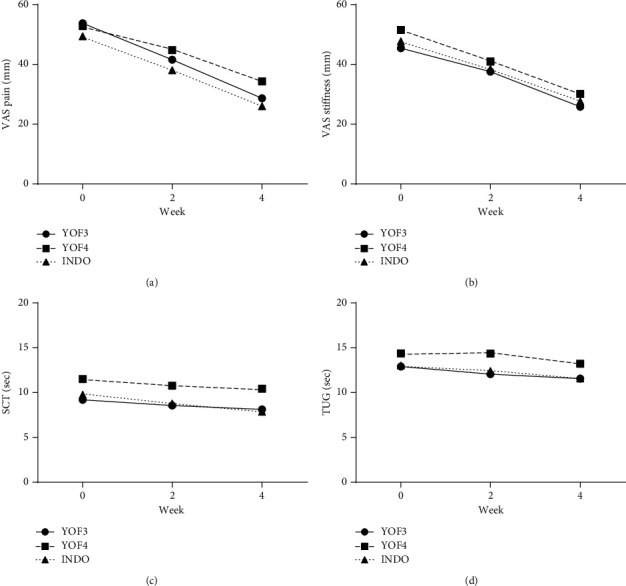
VAS pain (a), VAS stiffness (b), SCT (c), and TUG (d) at baseline, week 2, and week 4.

After receiving trial interventions for four weeks, the patients in all three groups had a statistically significant decrease in VAS pain, VAS stiffness, SCT value, and TUG value when compared with their respective baseline values (Figure 3; Table S2). VAS pain in the YOF3, YOF4, and INDO groups declined by 46.6%, 35.0%, and 47.3%, respectively, while VAS stiffness declined by 43.2%, 41.5%, and 42.0%, respectively. There were 15, 7, and 15 responders, contributing to 46.9%, 26.9%, and 46.9% response rates in the YOF3, YOF4, and INDO groups, respectively (*p* = 0.219). All subscales of KOOS were statistically significantly increased from the baseline values in each of the three groups with the exception of knee-related quality of life in the YOF4 group (Figure S1; Table S3).

**Figure 4:**
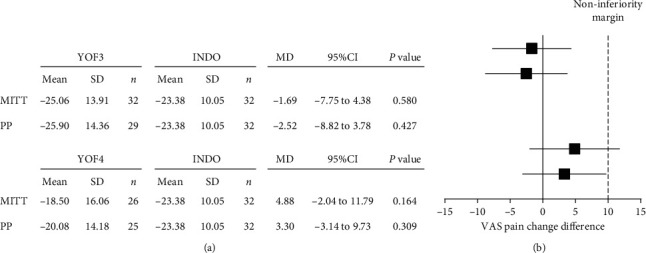
Noninferiority analysis of VAS pain.

No significant difference in mean changes of VAS pain, VAS stiffness, SCT value, TUG value, or any subscale of KOOS among the three groups was observed (Table 2). The average absolute reduction in VAS pain at the final visit was −25.06 ± 13.91, −18.50 ± 16.06, and −23.38 ± 10.05 mm in the YOF3, YOF4, and INDO groups, respectively (*p*=0.169). With regard to VAS pain, only YOF3 was found to be noninferior to INDO in both MITT and PP analyses, while YOF4 was not. The upper limit of two-sided 95% CI for the comparison between the YOF3 group and the INDO group was within the prespecified margin for noninferiority (Figure 4).

At the end of the trial, the patients rated their overall improvement at 64.59 ± 16.21, 50.77 ± 23.82, and 63.50 ± 22.86 in the YOF3, YOF4, and INDO groups, respectively (Figure S2). There was a significant difference between the YOF4 group and the INDO group (MD = −12.73, 95% CI = −25.24 to −.22, *p*=0.045). The physician's opinion of overall improvement at week 4 was 57.72 ± 23.44, 49.38 ± 31.68, and 66.97 ± 21.99 in the YOF3, YOF4, and INDO groups, respectively (Figure S2). As with patient-rated improvement, a significant difference was also seen in the physician's opinion between the YOF4 group and the INDO group (MD = −17.58, 95% CI = −32.81 to −2.35, *p*=0.045).

During the trial, most of the patients did not report any adverse events with the exception of three patients: one experienced a common cold (YOF4 group), one a shoulder dislocation (YOF4 group), and one a brain tumor (YOF3 group), all of which were considered to be unrelated to the trial intervention. No skin rash at the application site was observed in any of the three groups.

## 4. Discussion

Outcome assessment in this randomized controlled trial included both patient-reported and objective outcome measures suggested by OARSI [21]. The three core clinical measures, i.e., pain, physical function, and patient global assessment, were evaluated as patient-reported symptomatic outcomes. For objective outcome measurement, SCT and TUG tests were used to evaluate physical function. The study demonstrated that YOF3 was noninferior to INDO in reducing OA knee pain, while the efficacy of YOF4 against OA knee pain was inconclusive. The results provide support for the efficacy of YOF3 in the treatment of symptomatic OA of the knee. The topical application of YOF3 was as effective as INDO across all the measured outcome variables, and mean changes from baseline across the outcome parameters did not significantly differ between the two groups throughout the four-week study period. The relatively high dropout rate in the YOF4 group suggests that YOF4 might not be sufficiently effective for managing mild to moderate OA knee pain.

The absolute reduction in VAS pain after treatment for four weeks was −25.06 ± 13.91, −18.50 ± 16.06, and −23.38 ± 10.05 mm, in the YOF3, YOF4, and INDO groups, respectively, two of which (YOF3 and INDO) were below the minimal clinically important improvement level of −19.9 mm [34]. This finding suggests that the majority of patients in the YOF3 and INDO groups considered themselves clinically improved in terms of OA knee pain. This was also supported by categorical analysis of VAS pain, i.e., about half of the patients in the YOF3 and INDO groups were classified as responders with more than 50% pain reduction after treatment as compared with only one-fourth of the patients in the YOF4 group. A 50% decrease in pain score is commonly used to represent clinical meaningfulness of pain relief from the patient's perspective [33]. Based on the findings from the present trial, YOF3 may be considered an alternative therapy for pain management in knee OA patients with mild to moderate pain intensity.

The goals of treatment for OA of the knee are not only to relieve knee pain and inflammation but also to improve joint function, mobility, and the patient's quality of life. This trial assessed symptoms of knee OA using several different measures, providing a comprehensive evaluation of the efficacy of the trial interventions [35]. The values of two performance-based assessments, the SCT and TUG tests, were significantly reduced by about one to two seconds in all three groups on week 4, indicating an improvement in physical performance after treatment for four weeks. Moreover, all KOOS subscales were significantly increased in all three groups, suggesting that the physical function of the affected knee had been improved [36]. These favorable outcomes can be assumed to be due to pain and stiffness relief resulting from the intervention.

Although the exact mechanisms of action of YOF3 and YOF4 have not yet been elucidated, it is reasonable to postulate that herbal materials in the formulations mainly act via several pathways, including inhibition of cyclooxygenase (COX) and/or lipoxygenase (LOX), as well as inhibition of cytokine release [37]. In vitro and in vivo studies have demonstrated that *Z*. *montanum* Koenig exhibits potent anti-inflammatory activity through inhibition of the COX and LOX pathways [38, 39] and that it also shows chondroprotective activity [40]. A previous randomized clinical trial demonstrated the efficacy of *Z. montanum* Koenig cream in decreasing pain and improving functional ability in patients with a mild to moderate degree of knee OA [41]. Previous experiments have shown that the extract and essential oil of *S. aromaticum* Merr. et Perry possess anti-inflammatory properties which act through the inhibition of mRNA expression of COX-2 and inhibition of cytokine production through the suppression of the nuclear transcription factor kappa B (NF-*κ*B) pathway [42, 43]. Other evidence suggests that the essential oil from the bark of *C*. *aromaticum* Nees possesses antinociceptive and anti-inflammatory properties which act by blocking protein expression of inducible nitric oxide synthase (iNOS), COX-2, and NF-*κ*B [44, 45]. Menthol, an extract from *Mentha piperata* Linn., can also suppress the production of inflammatory mediators (e.g., leukotriene B and prostaglandin E (PGE)), in human monocytes [46]. Racemic camphor (*Cinnamonum camphora*) has been demonstrated to have anti-inflammatory activity through the modulation of cytokine, nitric oxide (NO), and PGE production in lipopolysaccharide (LPS)/interferon (IFN) gamma-activated macrophages [47], while borneol has been shown to produce anti-inflammatory and antinociceptive effects in animal experiments [48]. Various lines of evidence suggest that sesame oil possesses anti-inflammatory, antinociceptive, and chondroprotective properties as has been consistently shown in both nonclinical and human studies [49, 50]. The broad mechanisms of action of herbal materials in YOF3 may synergistically contribute to the beneficial effects of topical application of YOF3 in patients with symptomatic OA of the knee.

The present trial had some limitations associated with the single-blind, active-controlled, noninferiority design. In herbal drug trials, it has frequently proven impracticable or infeasible to provide indistinguishable preparations of the test drug and its comparator(s) [51]. Although indomethacin solution was more or less similar to yellow oil formulations, they were not alike. This may jeopardize the trial's blind assignment and introduce bias into the study. Although both outcome assessors and patients were not informed of the treatment allocation, color differences among the three formulations might have made the patients aware of what formulation they and the other patients were assigned in the present trial. Though VAS is valid and reliable for use in pain assessment, it is subjective and largely dependent on the patient's perception of pain [52, 53]. The literature suggests that the favorable effects of treatment interventions in OA knee trials may partly be attributable to contextual effects such as patient beliefs and expectancy as well as to the patient-physician relationship [54, 55]. In addition, withholding effective treatment might not be considered ethical in a setting where topical NSAIDs are available and widely prescribed [22]. We rather sought to determine whether yellow oil formulations are not inferior to a reference treatment (i.e., topical NSAIDs) by more than an acceptable amount.

The favorable outcomes from YOF3 should not be extrapolated beyond the four week period observed in this trial. The short-term benefit of the intervention is indeed consistent with several previous studies showing the efficacy of topical agents over the first two to four weeks [24, 56]. However, the current evidence does not support the use of topical agents, including topical NSAIDs, for long-term pain control in patients with OA of the knee [57, 58]. Further studies are required to assess the potential long-term efficacy and safety of YOF3 and/or YOF4 in patients with OA of the knee as this chronic and progressive disease requires long-term pharmacological management.

## 5. Conclusions

YOF3 applied four times daily was shown to be noninferior to INDO in relieving OA knee pain in this randomized controlled trial. YOF3 may be considered a reasonable alternative or supplementary for the treatment of symptomatic OA of the knee. Further research into the mechanism of action of YOF3 is warranted to better understand and elucidate its role in symptom relief of knee OA. It is also necessary to confirm its efficacy and safety in a larger and more definitive trial with a longer duration of follow-up.

## Figures and Tables

**Table 1:** Characteristics of study participants.

	YOF3 (*n* = 34)	YOF4 (*n* = 34)	INDO (*n* = 34)
Gender (female: male)	32 : 2	30 : 4	31 : 3
Age (years)^1^	62.29 ± 7.89	62.18 ± 6.77	60.76 ± 7.03
BMI (kg/m^2^)^1^	25.56 ± 3.73	25.86 ± 3.91	27.51 ± 4.81
Localization of OA (right knee: left knee: both knees)	5 : 5: 24	1 : 6: 27	2 : 5: 27
Kellgren and Lawrence X-ray grade (Grade 2: Grade 3: Grade 4)	20 : 23: 15	22 : 20: 19	29 : 15: 17
VAS pain^1^	53.65 ± 8.03	52.71 ± 9.45	49.24 ± 9.61
VAS stiffness^1^	45.79 ± 14.11	50.53 ± 15.40	47.76 ± 15.02
KOOS^1^			
Pain	53.59 ± 13.62	57.00 ± 13.29	53.68 ± 15.54
Other knee symptoms	58.91 ± 13.56	63.21 ± 15.57	58.38 ± 14.05
Activities of daily living	54.12 ± 13.88	55.26 ± 15.84	55.76 ± 17.05
Sport and recreation function	30.00 ± 16.24	25.59 ± 19.84	27.94 ± 19.70
Knee-related quality of life	33.29 ± 16.81	32.71 ± 13.66	32.47 ± 15.80

^1^Data shown as mean ± SD.

**Table 2:** Efficacy outcome assessment.

	YOF3	YOF4	INDO	*p* value^1^
*Mean change of VAS pain*				
MITT analysis	−25.06 ± 13.91	−18.50 ± 16.06	−23.38 ± 10.05	0.169
PP analysis	−25.90 ± 14.36	−20.08 ± 14.18	−23.38 ± 10.05	0.259

*Mean change of VAS stiffness*				
MITT analysis	−19.59 ± 11.74	−21.38 ± 16.05	−20.03 ± 13.86	0.881
PP analysis	−20.45 ± 11.87	−23.76 ± 10.75	−20.03 ± 13.86	0.484

*Mean change of time taken to climb up 10 steps*				
MITT analysis	−1.06 ± 2.45	−1.08 ± 1.74	−1.88 ± 2.34	0.264
PP analysis	−1.03 ± 2.43	−1.16 ± 1.72	−1.88 ± 2.34	0.284

*Mean change of time up and go test*				
MITT analysis	−1.34 ± 1.83	−1.15 ± 1.95	−1.44 ± 1.72	0.839
PP analysis	−1.28 ± 1.89	−1.32 ± 1.80	−1.44 ± 1.72	0.936

*Mean change of KOOS pain*				
MITT analysis	13.78 ± 12.56	9.69 ± 14.91	13.81 ± 14.64	0.456
PP analysis	14.59 ± 12.91	11.28 ± 12.78	13.81 ± 14.64	0.651

*Mean change of KOOS other symptoms*				
MITT analysis	10.75 ± 12.02	7.23 ± 14.64	13.13 ± 11.77	0.220
PP analysis	10.14 ± 12.24	8.08 ± 14.27	13.13 ± 11.77	0.324

*Mean change of KOOS activities of daily living*				
MITT analysis	11.53 ± 12.88	9.54 ± 16.64	12.91 ± 14.07	0.678
PP analysis	11.59 ± 13.53	11.32 ± 14.22	12.91 ± 14.07	0.896

*Mean change of KOOS sport and recreation function*				
MITT analysis	11.41 ± 15.72	12.69 ± 17.62	13.13 ± 16.35	0.911
PP analysis	11.38 ± 16.47	13.60 ± 17.35	13.13 ± 16.35	0.872

*Mean change of KOOS knee-related quality of life*				
MITT analysis	8.31 ± 13.57	4.81 ± 18.14	9.16 ± 13.21	0.516
PP analysis	7.69 ± 13.89	6.24 ± 16.95	9.16 ± 13.21	0.755

Data shown as mean ± SD. ^1^One-way ANOVA.

## Data Availability

All data used to support the findings of this study are available from the corresponding author upon reasonable request.

## Supplementary Materials

Supplementary Materials Figure S1: KOOS at baseline, week 2, and week 4. Figure S2: patient's and physician's opinion of overall improvement. Table S1 : components of YOF3 and YOF4. Table S2: VAS pain, VAS stiffness, SCT, and TUG at baseline, week 2, and week 4. Table S3: KOOS at baseline, week 2, and week 4.
