# Supplementary material for: Clinical Efficacy and Safety of Yellow Oil Formulations 3 and 4 versus Indomethacin Solution in Patients with Symptomatic Osteoarthritis of the Knee: A Randomized Controlled Trial
Source: Evid Based Complement Alternat Med. 2020 Jul 25;2020:5782178. doi: 10.1155/2020/5782178 (PMC7397436; doi:10.1155/2020/5782178)
Supplement: Supplementary Materials — Figure S1: KOOS at baseline, week 2, and week 4. Figure S2: patient's and physician's opinion of overall improvement. Table S1 : components of YOF3 and YOF4. Table S2: VAS pain, VAS stiffness, SCT, and TUG at baseline, week 2, and week 4. Table S3: KOOS at baseline, week 2, and week 4. [file 5782178.f1.zip › 5782178.f1/Table S1. Components of YOF3 and YOF4.pdf]

Table S1(A): Components of yellow oil formulation 3 (YOF3).

	Botanical/chemical name	Common name	Part used	Amount
1.	*Zingiber montanum* (Koenig)	Phlai	Rhizome	95%
2.	*Syzygium aromaticum* Merr. et Perry	Clove	Flower	
3.	*Cinnamomum aromaticum* Nees	Cinnamon	Bark	
4.	Menthol	Menthol		
5.	Racemic camphor	Racemic camphor		
6.	Borneal	Borneal		
7.	*Sesamum indicum* L.	Sesame	Seed oil	5%

Table S1(B): Components of yellow oil formulation 4 (YOF4).

	Botanical/chemical name	Common name	Part used	Amount
1.	*Zingiber montanum* (Koenig)	Phlai	Rhizome	90%
2.	*Curcuma longa* L.	Turmeric	Rhizome	
3.	*Syzygium aromaticum* Merr. et Perry	Clove	Flower	
4.	*Cocos nucifera* Linn.	Coconut	Oil	
5.	Menthol	Menthol		
6.	Racemic camphor	Racemic camphor		
7.	Borneol	Borneol		
8.	Methyl salicylate	Methyl salicylate	Essential oil	10%
