# Supplementary material for: Clinical Efficacy and Safety of Yellow Oil Formulations 3 and 4 versus Indomethacin Solution in Patients with Symptomatic Osteoarthritis of the Knee: A Randomized Controlled Trial
Source: Evid Based Complement Alternat Med. 2020 Jul 25;2020:5782178. doi: 10.1155/2020/5782178 (PMC7397436; doi:10.1155/2020/5782178)
Supplement: Supplementary Materials — Figure S1: KOOS at baseline, week 2, and week 4. Figure S2: patient's and physician's opinion of overall improvement. Table S1 : components of YOF3 and YOF4. Table S2: VAS pain, VAS stiffness, SCT, and TUG at baseline, week 2, and week 4. Table S3: KOOS at baseline, week 2, and week 4. [file 5782178.f1.zip › 5782178.f1/Table S2 VAS pain, VAS stiffness, SCT, TUG_revision .pdf]

Table S2. VAS pain, VAS stiffness, SCT, and TUG at baseline, week 2, and week 4.

	**YOF3**	**YOF4**	**INDO**
VAS pain (mm)			
MITT analysis			
Baseline	53.75 ± 8.17	52.81 ± 9.89	49.41 ± 9.85
Week 2	41.59 ± 11.55*	44.77 ± 13.27*	38.09 ± 14.59*
Week 4	28.69 ± 14.02*	34.31 ± 16.03*	26.03 ± 14.17*
PP analysis			
Baseline	54.10 ± 8.52	53.08 ± 9.99	49.41 ± 9.85
Week 2	42.45 ± 11.79*	43.88 ± 12.73*	38.09 ± 14.59*
Week 4	28.21 ± 14.65*	33.00 ± 14.88*	26.03 ± 14.17*
VAS stiffness (mm)			
MITT analysis			
Baseline	45.41 ± 14.41	51.50 ± 14.99	47.72 ± 15.49
Week 2	37.56 ± 14.87*	40.96 ± 16.65*	38.50 ± 18.55*
Week 4	25.81 ± 11.61*	30.12 ± 15.80*	27.69 ± 18.70*
PP analysis			
Baseline	47.03 ± 13.68	52.40 ± 14.56	47.72 ± 15.49
Week 2	39.55 ± 13.82*	39.92 ± 16.10*	38.50 ± 18.55*
Week 4	26.59 ± 11.51*	28.64 ± 14.18*	27.69 ± 18.70*
SCT (sec)			
MITT analysis			
Baseline	9.19 ± 3.09	11.50 ± 5.35	9.75 ± 3.38
Week 2	8.56 ± 2.14	10.77 ± 5.16*	8.78 ± 3.03*
Week 4	8.13 ± 2.09*	10.42 ± 5.25*,**	7.88 ± 2.15*
PP analysis			
Baseline	9.17 ± 3.19	10.96 ± 4.69	9.75 ± 3.38
Week 2	8.62 ± 2.23	10.16 ± 4.20*	8.78 ± 3.03*
Week 4	8.14 ± 2.18*	9.80 ± 4.26*,**	7.88 ± 2.15*
TUG (sec)			
MITT analysis			
Baseline	12.91 ± 2.63	14.38 ± 4.62	13.03 ± 2.71
Week 2	12.06 ± 2.33*	14.35 ± 5.56	12.41 ± 2.92
Week 4	11.56 ± 1.95*	13.23 ± 4.91*	11.59 ± 1.97*
PP analysis			
Baseline	12.86 ± 2.60	13.84 ± 3.77	13.03 ± 2.71
Week 2	12.14 ± 2.34	13.68 ± 4.49	12.41 ± 2.92
Week 4	11.59 ± 1.94*	12.52 ± 3.38*	11.59 ± 1.97*

Data represent mean ± SD. *p <0.05 versus baseline value (one-way repeated measures ANOVA, followed by LSD test). **p <0.05 versus INDO (one-way ANOVA, followed by the Dunnett test).
