# Supplementary material for: Clinical Efficacy and Safety of Yellow Oil Formulations 3 and 4 versus Indomethacin Solution in Patients with Symptomatic Osteoarthritis of the Knee: A Randomized Controlled Trial
Source: Evid Based Complement Alternat Med. 2020 Jul 25;2020:5782178. doi: 10.1155/2020/5782178 (PMC7397436; doi:10.1155/2020/5782178)
Supplement: Supplementary Materials — Figure S1: KOOS at baseline, week 2, and week 4. Figure S2: patient's and physician's opinion of overall improvement. Table S1 : components of YOF3 and YOF4. Table S2: VAS pain, VAS stiffness, SCT, and TUG at baseline, week 2, and week 4. Table S3: KOOS at baseline, week 2, and week 4. [file 5782178.f1.zip › 5782178.f1/Table S3 KOOS_revision ver1.1.pdf]

Table S3: KOOS at baseline, week 2, and week 4

	**YOF3**	**YOF4**	**INDO**
Pain			
MITT analysis			
Baseline	53.38 ± 13.99	56.92 ± 13.20	52.75 ± 15.23
Week 2	61.03 ± 14.12*	60.73 ± 16.20	61.00 ± 16.01*
Week 4	67.16 ± 14.27*	66.62 ± 13.97*	66.56 ± 16.40*
PP analysis			
Baseline	52.41 ± 14.28	56.32 ± 13.10	52.75 ± 15.23
Week 2	60.24 ± 14.44*	61.48 ± 16.07	61.00 ± 16.01*
Week 4	67.00 ± 14.82*	67.60 ± 13.31*	66.56 ± 16.40*
Symptoms			
MITT analysis			
Baseline	58.38 ± 13.78	62.54 ± 14.58	57.22 ± 13.03
Week 2	65.06 ± 14.50*	68.31 ± 15.50	65.00 ± 15.74*
Week 4	69.13 ± 16.31*	69.77 ± 13.25*	70.34 ± 13.70*
PP analysis			
Baseline	58.66 ± 14.27	62.32 ± 14.83	57.22 ± 13.03
Week 2	64.31 ± 15.04*	68.88 ± 15.53	65.00 ± 15.74*
Week 4	68.79 ± 17.12*	70.40 ± 13.12*	70.34 ± 13.70*
Activities of daily living			
MITT analysis			
Baseline	53.88 ± 14.28	55.88 ± 16.18	54.03 ± 15.84
Week 2	61.34 ± 14.03*	61.08 ± 15.65	61.25 ± 15.06*
Week 4	65.41 ± 14.73*	65.42 ± 15.99*	66.94 ± 14.62*
PP analysis			
Baseline	53.38 ± 14.52	55.24 ± 16.17	54.03 ± 15.84
Week 2	60.48 ± 14.06*	62.04 ± 15.16*	61.25 ± 15.06*
Week 4	64.97 ± 15.03*	66.58 ± 15.20*	66.94 ± 14.62*
Sport and recreation function			
MITT analysis			
Baseline	28.59 ± 15.04	25.19 ± 21.00	28.44 ± 20.22
Week 2	37.50 ± 11.71*	33.65 ± 22.74*	29.53 ± 18.72
Week 4	40.00 ± 16.11*	37.88 ± 21.36*	41.56 ± 22.27*
PP analysis			
Baseline	27.41 ± 15.33	25.80 ± 21.20	28.44 ± 20.22
Week 2	36.03 ± 11.21*	35.00 ± 22.13*	29.53 ± 18.72
Week 4	38.79 ± 16.40*	39.40 ± 20.33*	41.56 ± 22.27*
Knee-related quality of life			
MITT analysis			
Baseline	32.44 ± 16.95	33.35 ± 12.61	31.75 ± 15.95
Week 2	38.00 ± 14.44*	29.96 ± 14.86	36.63 ± 19.90

Week 4	40.75 ± 17.35*	38.15 ± 17.05	40.91 ± 18.75*
PP analysis			
Baseline	32.97 ± 17.41	32.92 ± 12.68	31.75 ± 15.95
Week 2	37.62 ± 14.18	30.64 ± 14.75	36.63 ± 19.90
Week 4	40.66 ± 17.46*	39.16 ± 16.59	40.91 ± 18.75*

Data represent mean ± SD. $^{*}p < 0.05$ versus baseline value (one-way repeated measures ANOVA, followed by LSD test). No statistically significant difference was found between YOF3 or YOF4 versus INDO (one-way ANOVA, followed by the Dunnett test).
