# Supplementary figures and images for: Clinical Efficacy and Safety of Yellow Oil Formulations 3 and 4 versus Indomethacin Solution in Patients with Symptomatic Osteoarthritis of the Knee: A Randomized Controlled Trial
Source: Evid Based Complement Alternat Med. 2020 Jul 25;2020:5782178. doi: 10.1155/2020/5782178 (PMC7397436; doi:10.1155/2020/5782178)

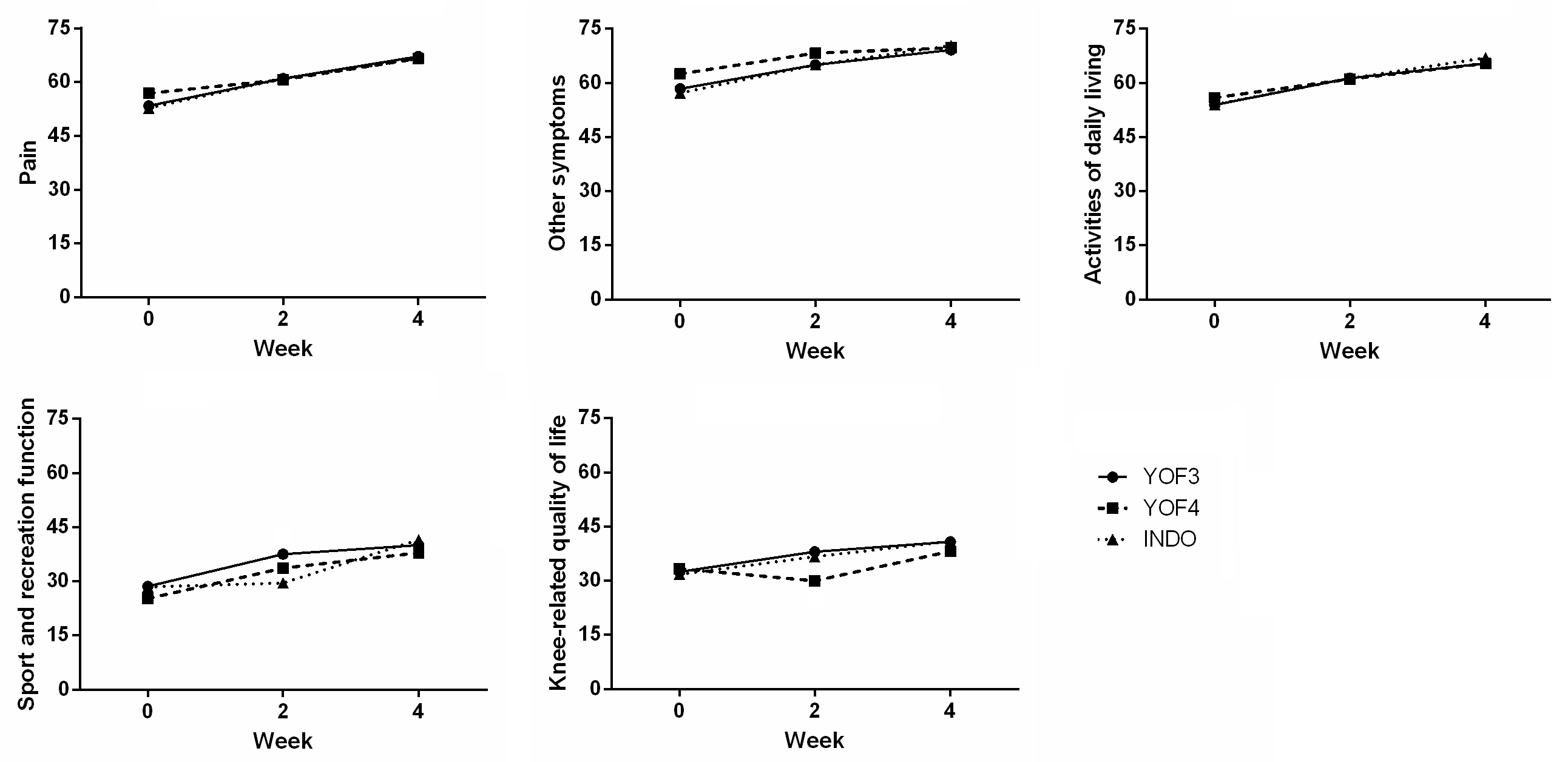

Supplement: Supplementary Materials — Figure S1: KOOS at baseline, week 2, and week 4. Figure S2: patient's and physician's opinion of overall improvement. Table S1 : components of YOF3 and YOF4. Table S2: VAS pain, VAS stiffness, SCT, and TUG at baseline, week 2, and week 4. Table S3: KOOS at baseline, week 2, and week 4. [file 5782178.f1.zip › 5782178.f1/Fig. S1 KOOS_revision.tif]

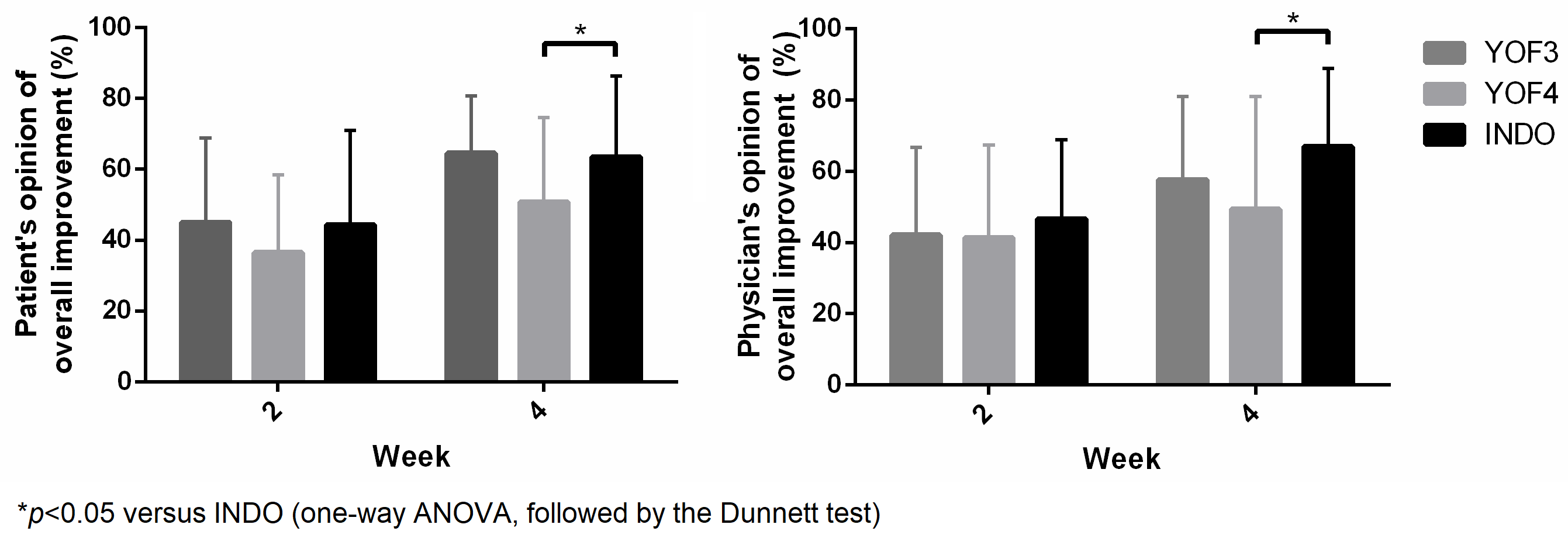

Supplement: Supplementary Materials — Figure S1: KOOS at baseline, week 2, and week 4. Figure S2: patient's and physician's opinion of overall improvement. Table S1 : components of YOF3 and YOF4. Table S2: VAS pain, VAS stiffness, SCT, and TUG at baseline, week 2, and week 4. Table S3: KOOS at baseline, week 2, and week 4. [file 5782178.f1.zip › 5782178.f1/Fig. S2 overall opinion_revision.tif]
